# treestructure: an R package to detect population structure in phylogenetic trees

**DOI:** 10.1093/bioinformatics/btag195

**Published:** 2026-04-22

**Authors:** Fabrícia F Nascimento, Vinicius B Franceschi, Erik M Volz

**Affiliations:** MRC Centre for Global Infectious Disease Analysis, Department of Infectious Disease Epidemiology, School of Public Health, Imperial College London, London, W12 0BZ, United Kingdom; MRC Centre for Global Infectious Disease Analysis, Department of Infectious Disease Epidemiology, School of Public Health, Imperial College London, London, W12 0BZ, United Kingdom; MRC Centre for Global Infectious Disease Analysis, Department of Infectious Disease Epidemiology, School of Public Health, Imperial College London, London, W12 0BZ, United Kingdom

## Abstract

**Motivation:**

How population structure can shape genetic diversity is a longstanding problem in population genetics. While the use of geographic locations, when available, can help answer some of these questions, it is still difficult to determine population structure when such metadata are not available or when the potential population structure is not easily observed. Here, we present an updated version of *treestructure*, an R package that implements a statistical test based on coalescent theory to detect unobserved population structure in a time-scaled phylogenetic tree.

**Availability:**

treestructure is available at CRAN at https://cloud.r-project.org/web/packages/treestructure/ and at https://emvolz-phylodynamics.github.io/treestructure/.

## 1 Introduction

The *treestructure* R package implements statistical tests based on coalescent theory to detect unobserved population structure in time-scaled phylogenetic trees (Volz et al. 2020). A time-scaled phylogenetic tree shows the evolutionary relationships between organisms in units of calendar time, and *treestructure* will detect clusters that are likely to be the result of distinct population structure with heterogeneous demographic or epidemiological history (Volz et al. 2020). The algorithm implemented in *treestructure* also groups clusters showing similar population structure into partitions. The initial version of the package has been used in a variety of studies, such as the detection of lineages with different demographic histories in SARS-CoV-2 (Fountain-Jones et al. 2020); the detection of a fitness advantage in clades that showed similar demographic histories in *Neisseria gonorrhoea* (Joseph et al. 2022); and the understanding of population of *Vibrio parahaemolyticus* in Latin America (Campbell et al. 2024).

In the new version of *treestructure*, we have added new functionalities that have not been previously described, enhancing its practical utility and statistical robustness. These new functionalities include (i) methods to automatically choose clustering hyperparameters, (ii) use of branch support values (e.g. bootstrap and posterior clade credibility) to filter out clusters with low phylogenetic confidence, and (iii) the addition of new tips to a previous *treestructure* object, allowing clusters to be updated in an online fashion as new data become available.

Other methods have been developed to detect population structure in phylogenetic trees, such as *CaveDive* (Helekal et al. 2022) and *fastbaps* (Tonkin-Hill et al. 2019), and have been applied to the detection of outbreaks and variant surveillance (Reimche et al. 2023, Binney et al. 2025). However, none of these methods can take into consideration the use of branch support to avoid detecting clusters on unsupported clades.

## 2 Implementation

*treestructure* is implemented as an R (R Core Team 2025) package and is available on CRAN (https://cloud.r-project.org/web/packages/treestructure/) and on GitHub (https://github.com/emvolz-phylodynamics/treestructure). For complete details on installation, documentation and tutorials using the new features, see the package website (https://emvolz-phylodynamics.github.io/treestructure).

Below, we give details on the new features and how these enhance the practical utility and statistical robustness of *treestructure* by using a publicly available time-scaled phylogeny based on Ebola virus whole genomes (https://github.com/ebov/space-time/blob/master/Data/Makona_1610_cds_ig.GLM.MCC.tree) collected during the 2014 epidemic in West Africa (Dudas et al. 2017).

### 2.1 Clustering significance level

Clustering methods, such as the one used in *treestructure*, require the specification of hyperparameters that specify how aggressively a method will partition data. The *treestructure* algorithm repeatedly tests subclades for deviations in their time of the most recent common ancestor (TMRCA) distributions from expectations under a neutral coalescent model and divides the tree where such deviations occur. To perform this test, it uses a *rank-sum* statistic, and clusters are defined when this coalescent-based statistical test detects a difference according to a desired significance level. Decreasing the significance level in the *treestructure* algorithm will increase the number of clusters detected. However, detecting more clusters will also increase the number of false-positive detections.

To determine the significance level, users can use additional metadata associated with each sample and then select the significance level which gives a set of clusters that explains the most variance in the data of interest (e.g. use the cluster as a factor in an ANOVA).

If metadata information is not available, users can use the new feature that implements the Caliński–Harabasz index, or CH-index (Caliński and Harabasz 1974) which is a metric based on within- and between-cluster variance in a given statistic to select a quasi-optimal significance level. Within *treestructure* we use the node heights of the phylogeny itself, observed within each cluster, as the statistic that is used when computing the CH-index. Thus, clusters are selected such that there is high between-cluster variance in phylogenetic node heights. A step-by-step tutorial on how to run such an analysis can be found on the *treestructure* website (https://emvolz-phylodynamics.github.io/treestructure/articles/supportValues.html).

### 2.2 Branch support values

As there is often a great deal of uncertainty about individual phylogenetic splits, a user may not want to cluster their data along branches which are poorly supported. Because of that, we have implemented the use of branch support (e.g. bootstrap and posterior probability) to refine clusters in *treestructure*. To use this functionality, the time-scaled tree should be annotated with node support values, and the user will need to define a node support threshold value between 0 and 100. Nodes with support values less than the threshold value will not be tested. This feature is very useful for filtering out clusters that may not correspond to real phylogenetic splits, which was the case when analysing HIV-1 sequences published by our group (Franceschi et al. 2025).

**Figure 1:**
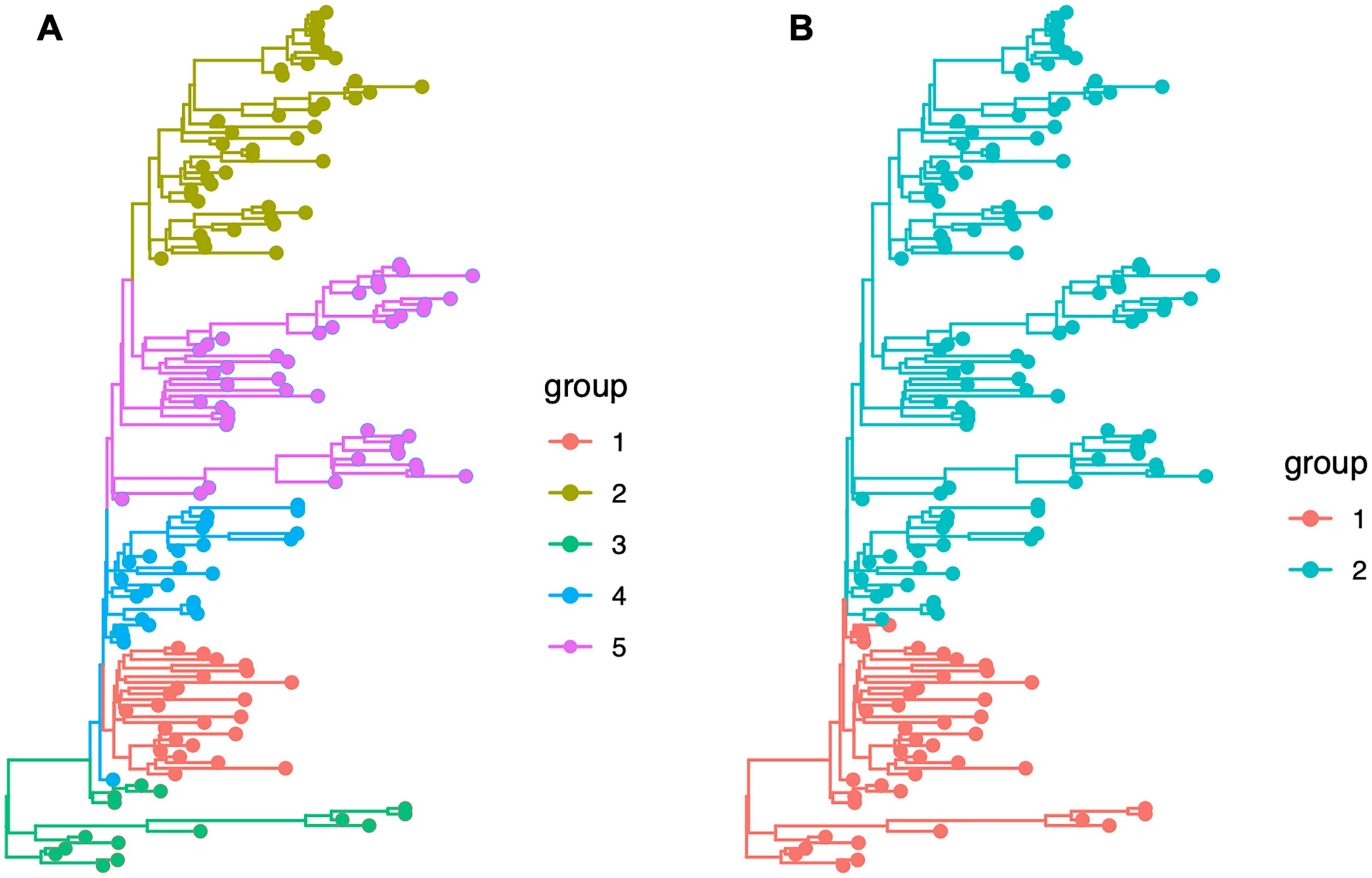
Clusters obtained by running *treestructure*, (A) without the use of branch support and (B) using a branch support threshold of 95. For both analyses, we used a significance level of 0.01 and a minimum clade size of 15 sequences. This figure was created using a publicly available Ebola time-scaled phylogenetic tree (https://github.com/ebov/space-time/blob/master/Data/Makona_1610_cds_ig.GLM.MCC.tree) downsampled to 150 sequences. An example analysing the complete Ebola dataset with 1610 sequences can be found on the *treestructure* website (https://emvolz-phylodynamics.github.io/treestructure/articles/supportValues.html).

Figure 1 shows an example of how the use of node support can filter out clusters with low phylogenetic confidence. Note that in the previous description of *treestructure*, the researcher would observe a higher number of clusters, many of which are in poorly supported clades as observed in this downsampled Ebola data (Fig. 1A).

### 2.3 Online inference by adding new samples to a previous *treestructure* object

Without the need to run multiple *treestructure* analyses, users can now update a *treestructure* object with new samples observed in a phylogenetic tree. This is particularly useful for classifying the cluster type of new samples without re-estimating and reassigning cluster membership. The updated tree does not need to be time-scaled or binary, reducing the need for expensive computation.

This new feature is implemented in the *addtips* function in the *treestructure* R package. The function *addtips* will compare the new phylogenetic tree to the old *treestructure* object, and it will merge the tips of the new tree into the *treestructure* object. Merging is carried out based on a phylogenetic criterion: New tips are added to the cluster which includes their most recent common ancestor in the new phylogeny. A step-by-step tutorial on how to use this feature can be found at https://emvolz-phylodynamics.github.io/treestructure/articles/updating_treestructure.html.

## Data Availability

Data and scripts used in this manuscript are available at https://github.com/emvolz-phylodynamics/treestructure/ Ebola phylogenetic tree used to generate Fig. 1 is available at https://raw.githubusercontent.com/ebov/space-time/master/Data/Makona_1610_cds_ig.GLM.MCC.tree
